# Oncogenic lncRNA ZNF561-AS1 is essential for colorectal cancer proliferation and survival through regulation of miR-26a-3p/miR-128-5p-SRSF6 axis

**DOI:** 10.1186/s13046-021-01882-1

**Published:** 2021-02-23

**Authors:** Zizhen Si, Lei Yu, Haoyu Jing, Lun Wu, Xidi Wang

**Affiliations:** 1 Pharmacy Department, The Affiliated Hospital of Medical School, Ningbo University, Ningbo, People’s Republic of China; 2 Department of Physiology and Pharmacology, Ningbo University School of Medicine, Ningbo, People’s Republic of China; 3 Department of Colorectal Cancer Surgery, The Second Affiliated Hospital of Harbin Medical University, Harbin, 150086 People’s Republic of China; 4 Department of Biochemistry and Molecular Biology, Harbin Medical University, 194 XueFu Road Nangang Dist, Harbin, 150086 People’s Republic of China

**Keywords:** Colorectal cancer, ZNF561-AS1, SRSF6, ceRNA

## Abstract

**Background:**

Long non-coding RNAs (lncRNA) are reported to influence colorectal cancer (CRC) progression. Currently, the functions of the lncRNA ZNF561 antisense RNA 1 (ZNF561-AS1) in CRC are unknown.

**Methods:**

ZNF561-AS1 and SRSF6 expression in CRC patient samples and CRC cell lines was evaluated through TCGA database analysis, western blot along with real-time PCR. SRSF6 expression in CRC cells was also examined upon ZNF561-AS1 depletion or overexpression. Interaction between miR-26a-3p, miR-128-5p, ZNF561-AS1, and SRSF6 was examined by dual luciferase reporter assay, as well as RNA binding protein immunoprecipitation (RIP) assay. Small interfering RNA (siRNA) mediated knockdown experiments were performed to assess the role of ZNF561-AS1 and SRSF6 in the proliferative actives and apoptosis rate of CRC cells. A mouse xenograft model was employed to assess tumor growth upon ZNF561-AS1 knockdown and SRSF6 rescue.

**Results:**

We find that ZNF561-AS1 and SRSF6 were upregulated in CRC patient tissues. ZNF561-AS1 expression was reduced in tissues from treated CRC patients but upregulated in CRC tissues from relapsed patients. SRSF6 expression was suppressed and enhanced by ZNF561-AS1 depletion and overexpression, respectively. Mechanistically, ZNF561-AS1 regulated SRSF6 expression by sponging miR-26a-3p and miR-128-5p. ZNF561-AS1-miR-26a-3p/miR-128-5p-SRSF6 axis was required for CRC proliferation and survival. ZNF561-AS1 knockdown suppressed CRC cell proliferation and triggered apoptosis. ZNF561-AS1 depletion suppressed the growth of tumors in a model of a nude mouse xenograft. Similar observations were made upon SRSF6 depletion. SRSF6 overexpression reversed the inhibitory activities of ZNF561-AS1 in vivo, as well as in vitro.

**Conclusion:**

In summary, we find that ZNF561-AS1 promotes CRC progression via the miR-26a-3p/miR-128-5p-SRSF6 axis. This study reveals new perspectives into the role of ZNF561-AS1 in CRC.

**Supplementary Information:**

The online version contains supplementary material available at 10.1186/s13046-021-01882-1.

## Background

Colorectal cancer (CRC) ranks 3rd among the most frequent cancers globally [1]. Despite remarkable advances, the molecular basis of CRC progression is not fully understood and more effective therapeutic strategies are needed [2].

Long non-coding RNAs (lncRNAs) are composed of > 200 nucleotides reported to influence various disorders [3–5], including cancers [6–8], by adsorbing miRNAs [9], mediating DNA interactions [10], and binding to proteins as decoys [11]. Many lncRNAs are show abnormal expression in CRC which affects its progression and drug resistance [12–14]. For instance, lncRNA GLCC1 promotes CRC tumorigenesis by stabilizing c-Myc [15]. By regulating the activities of PKM2, actions of FEZF1-AS1 leads to accelerated cell proliferation and metastasis [16]. Exosomal lncRNA H19 confers CRC cells stemness and chemo-resistance [17]. Since lncRNAs influence CRC progression, their identification and understanding of their biological functions in CRC are imperative as they have anti-CRC therapeutic value.

ZNF561-AS1 is a novel lncRNA whose function in CRC is unknown. Here, we find that ZNF561-AS1 overexpressed in CRC samples and this promotes the proliferative rates and survival of CRC cells in vivo, as well as in vitro. Mechanically, ZNF561-AS1 upregulates SRSF6 levels by sponging miR-26a-3p and miR-128-5p. Additionally, reduced SRSF6 levels upon ZNF561-AS1 depletion were restored by miR-26a-3p or/and miR-128-5p inhibition. Exogenous SRSF6 expression rescued proliferation of ZNF561-AS1-depleted CRC cells. The present results underscores the potential of ZNF561-AS1 to be targeted as an anti-CRC target.

## Materials and methods

### Clinical tissues

Six human colorectal cancer tissues along with matched adjacent normal tissues were collected from the Second Affiliated Hospital of Harbin Medical University with patients’ consents. The ethics committee of Harbin Medical University approved the study. Matched pre-treated, post-treated and recurrent samples from one patient, in total six patients were collected. The samples obtained at surgery refers to pre-treated samples. Post-treatment samples were collected after finishing chemotherapy by colonoscope. In 1–2 years after chemotherapy, the patients were suffered from relapsed tumors which refer to recurrent samples. Upon collection, tissues were snap-frozen in liquid nitrogen for further use.

Patients information is shown on Supplementary Table S1.

### Cell culture

The following human CRC cell lines were used in the experiments: HCT-116, HT-29, SW620, LoVo, SW480, and SW48, all obtained from the Cell Bank of the Chinese Academy of Sciences (Shanghai, China). These cells were inoculated in Dulbecco’s modified Eagle medium (DMEM, Invitrogen, Carlsbad, CA, USA), containing 10% fetal bovine serum (FBS; Invitrogen), 50 μg/mL streptomycin and 50 U/mL penicillin (Invitrogen). CCD841 CoN, a normal human colonic epithelial cell line, was supplied by the American Type Culture Collection (ATCC, Manassas, VA, USA) and grown in Eagle’s Minimum Essential Medium (EMEM, Lonza, Basel, Switzerland), supplemented with 10% FBS. All cells were maintained in an incubator with humidified conditions at 37 °C, 5% CO_2_.

### Cell transfection

ZNF561-AS1 siRNA, as well as negative control siRNA were constructed by GenePharma, Shanghai, China. MiR-26a-5p and miR-128-3p mimics, inhibitor NC, mimics NC, miR-26a-5p and miR-128-3p inhibitors and were purchased from Ambion (Austin, TX, USA). ZNF561-AS1 and SRSF6 expression vectors were purchased from GeneCopoeia, Guangzhou, China. To transfect the cells with vectors, HCT-116 cells were first cultured in 6 cm dishes for 24 h. The cells were transfected using lipofectamine 2000 for 5 h, and then the transfection media was replaced with complete media. The transfected cells were harvested at 48 h after transfection.

### Measurement of mRNA expression of ZNF561-AS1

Total RNA for Real-time PCR was isolated by lysing the cells with TRIzol (Invitrogen,). cDNA was generated from the RNA using reverse transcription kit. Real time PCR for ZNF561-AS1 expression was performed following routine procedures using the Power SYBR Green PCR master Mix (Thermo Fisher Scientific, Waltham, USA). Relative mRNA expression was determined using the 2^-ΔΔCt^ method. Experiments were run in triplicate and average values were obtained. The primers used to measure ZNF561-AS1 expression were:

ZNF561-AS1-F: 5′- ACCAAGACCTCCCACAACTCTCC -3′;

ZNF561-AS1-R: 5′- CAGGATCTGGCTTCACTGCTCTTC -3′.

GAPDH-F: 5′- CTGGGCTACACTGAGCACC -3′;

GAPDH-R: 5′- AAGTGGTCGTTGAGGGCAATG -3′.

GAPDH was used as reference gene.

To assess miR-26a-5p and miR-128-3p expression levels, 1 μg of total RNA was retrotranscribed using miR-26a-5p or miR-128-3p specific stem-loop primers (Genepharma, Shanghai, China). RT-qPCR analysis of miR-26a-5p or miR-128-3p was done with the Power SYBR Green PCR master Mix. The stem loop primers and PCR primers for miR-26a-5p or miR-128-3p used in the reverse transcription and qPCR were:

miR-26a-5p specific stem loop primer: GTCGTATCCAGTGCGTGTCGTGGAGTCGGCAATTGCACTGGATACGACAGCCTATC.

miR-26a-5p-F: 5′- GGCTTCAAGTAATCCAGG − 3′.

miR-26a-5p-R: 5′- ATTGCGTGTCGTGGAGTCG − 3′.

miR-128-3p specific stem loop primer: GTCGTATCCAGTGCGTGTCGTGGAGTCGGCAATTGCACTGGATACGACTTTCTCTG.

miR-128-3p-F: 5′- GGGTCACAGTGAACCGGTC − 3′.

miR-128-3p-R: 5′- ATTGCGTGTCGTGGAGTCG -3′.

U6-F: 5′- GCTTCGGCAGCACATATACTAAAAT -3′.

U6-R: 5′- CGCTTCACGAATTTGCGTGTCAT − 3′.

U6 was served for normalization.

### Western blot analysis

A RIPA buffer supplemented with protease inhibitors cocktail (Roche, Switzerland) was used to extract protein samples from cells. Equal amounts of proteins were resolved on SDS-PAGE and electro-transferred to PVDF membranes (Thermo Scientific, USA) and then blocked with 5% skim mile. After blocking, the blots were probed with following primary antibodies to SRSF6 (1:1000, Abcam, Cambridge, MA, USA), PCNA (1:1000), Cyclin D1 (1:1000), CDK4 (1:1000), cleaved Caspase-3 (1:1000) and p21 (1:1000) (Cell signaling technology, USA) and Actin (1:1000, Santa Cruz, USA). After washing and inoculating with HRP-conjugated rabbit or mouse, secondary antibodies (1:5000, Cell signaling Technology), signal was then developed using ECL reagent (GE healthcare, USA).

### Cell viability assays

ZNF561-AS1 siRNA or control siRNA transfected HCT-116 cells were seeded in 96-well plates with 4 × 10^3^ cells in each well for 4 days. The viability of cells was evaluated using a Cell Counting Kit-8 (CCK-8, Dojindo, Japan) at day 0, 2, and 4.

### Colony formation assays

800 HCT-116 cells transfected with ZNF561-AS1 siRNA or control siRNA were counted and seeded into 6 cm dishes and cultured for 14 days. The cells were stained with 0.1% crystal violet in 20% methanol for 20 min, and the number of colonies formed were photographed and counted.

### Immunofluorescence staining

HCT-116 cells transfected with ZNF561-AS1 siRNA or control siRNA respectively were seeded onto coverslips. 48 h after transfection, cells were fixed with 4% PFA at room temperature. This was followed by incubation with mouse anti-Ki-67 primary antibody (1:100, Cell Signaling Technology, USA) at room temperature for 1 h. Then, cells were washed with PBS and incubated at room temperature for 20 min with of anti-mouse Alexa-488 secondary antibody (1:1000, Cell Signaling Technology, USA) and counterstained with DAPI. Stained coverslips were mounted using prolong® diamond antifade mountant (Applied Biosystems, USA). Images were captured using Zeiss Axiovert 200 microscope.

### Acridine orange/ethidium bromide (AO/EB) fluorescence staining

After transfection with ZNF561-AS1 siRNA or control siRNA for 48 h, HCT-116 cells were treated with AO/EB reagent (Solarbio Biotechnology, China) for 5 min. The morphology of cells after treatment was imaged with a fluorescence microscope at 200X magnification. The percentage of cell apoptosis was determined as follows: apoptotic rate(%) = number of apoptotic cells/number of all cells counted.

### TUNEL assay

Click-iT TUNEL Alexa Fluor Imaging Assay Kit (Invitrogen, USA) was employed to explore cells apoptosis. In brief, control siRNA or ZNF561-AS1 siRNA transfected HCT-116 cells were fixed with 4% PFA for 15 min and permeabilized with 0.25% Triton X-100 for 10 min at room temperature. Then cells were washed and incubated with TdT reaction buffer for 10 min and incubated with Click-iT reaction cocktail for 30 min at room temperature. Cell nuclei were counterstained with DAPI after washing with PBS. Stained coverslips were mounted using prolong® diamond antifade mountant (Applied Biosystems, USA). Cells were imaged using a Zeiss Axiovert 200 microscope.

### Nude mice experiments

Athymic BALB/c nude mice (6-weeks old) were purchased from Vital River Laboratory, Beijing, China to establish tumor xenograft models. Mice were maintained and housed under specific pathogen-free conditions and handled using aseptic procedures.

HCT-116 cells (5 × 10^6^) transfected with ZNF561-AS1 siRNA or control siRNA were then subcutaneously administered into the right shank of the mice (3 mice/group). Tumor size (length; L, and width; W) determined with a caliper every 7 days and tumor volume given by the formula: ½LW^2^. Three weeks later, mice were killed. Isolated xenograft tumors were weighed and subjected to the further experiments.

### Immunohistochemistry staining

Harvested tumors were fixed in 4% PFA for 24 h at room temperature, followed by permeabilization with PBST for 20 min and inactivation of endogenous peroxidases by incubating tissues in 0.3% H_2_O_2_ for 20 min. After washing, tissues were blocked with 5% normal goat serum (Invitrogen, USA) for 30 min and incubated with anti-Ki-67 antibody (Cell Signaling Technology, USA) overnight at 4 °C. Subsequently, tissues were rinsed and probed with a biotin-labeled secondary antibody for 30 min and washed. After washing, the specimens were developed with 0.05% DAB (Sigma-Aldrich, Oakville, ON, Canada) and 0.03% H2O2, and counterstained with hematoxylin, dehydrated in increasing ethanol concentrations, cleared with xylene. Images of stained specimens were captured using an Olympus BX51 microscope. Digital images were analyzed using Image-Pro Plus 6.0 software.

### RNA binding protein immunoprecipitation (RIP)

RIP assay was done using the EZ-Magna RIP™ RNA-Binding Protein Immunoprecipitation Kit (Millipore, Billerica, MA, USA) as described by the manufacturer protocol. Briefly, HCT-116 cells were lysed in complete RIP lysis buffer. 100 μl whole cell lysate from each group were incubated with RIP buffer containing magnetic beads conjugated to mouse anti-Ago2 antibody (Millipore, USA), or negative control normal mouse IgG (Millipore, USA). Next, proteins were digest by incubated with proteinase K and immunoprecipitated RNA was harvested. The RNA concentration was quantified on NanoDrop spectrophotometer (Thermo Scientific, USA). Purified RNA was reverse transcribed into cDNA and subjected to qPCR to examine the presence of ZNF561-AS1, miR-26a-3p, and miR-128-5p using indicated primers.

### Dual luciferase reporter assay

MiR-26a-3p and miR-128-5p binding sites on ZNF561-AS1 and SRSF6 were searched on bioinformatics platforms. Wild-type binding site and flanking sequences (~ 300 bp) were amplified and cloned into *Spe*I and *Hind*III sites of the pMIR-REPORT Luciferase vector (Ambion, Austin, TX, USA) and named ZNF561-AS1-WT and SRSF6-WT. To generate mutant miR-26a-3p and/or miR-128-5p binding sites in the luciferase vector, several point mutations were introduced into ZNF561-AS1-WT and SRSF6-WT using Phusion Site-Directed Mutagenesis Kit (Thermo Fisher Scientific, USA), and named ZNF561-AS1-26aMut or ZNF561-AS1-128Mut, and SRSF6-26aMut or SRSF6-128Mut (single mutant) or ZNF561-AS1-26a/128Mut or SRSF6-26a/128Mut (double mutant). To determine if ZNF561-AS1 and SRSF6 are direct miR-26a-3p and miR-128-3p targets, HCT-116 cells were seeded in 6-well plates and co-transfected with the indicated vectors using the Invitrogen Lipofectamine 2000 (Invitrogen, USA) for 48 h. Luciferase activity was analyzed using Promega Dual Luciferase-reporter 1000 assay system (Promega, Madison, WI, USA) and normalized to Renilla luciferase activity.

### Data analysis

For biological experiments, data were obtained from at least 3 independent experiments and presented as the mean ± SD. The data were subjected to F-test, and then subjected to two-tailed Student’s t test (comparison between two groups) or ANOVA (Comparisons among three groups or more) using Statistical Program for Social Sciences (SPSS) version 17.0 (Chicago, IL, USA). GEPIA web server was utilized to analyze the data of expression of ZNF561-AS1 and SRSF6 in CRC tumor and adjacent normal tissues from TCGA database. The data were subjected to ANOVA analysis for differentially expressed genes based on the comparison of tumors and matched normal samples, and Pearson correlation analysis for correlation between ZNF561-AS1 and SRSF6.

## Results

### Overexpression of ZNF561-AS1 in CRC positively regulates SRSF6 expression

**Fig. 1:**
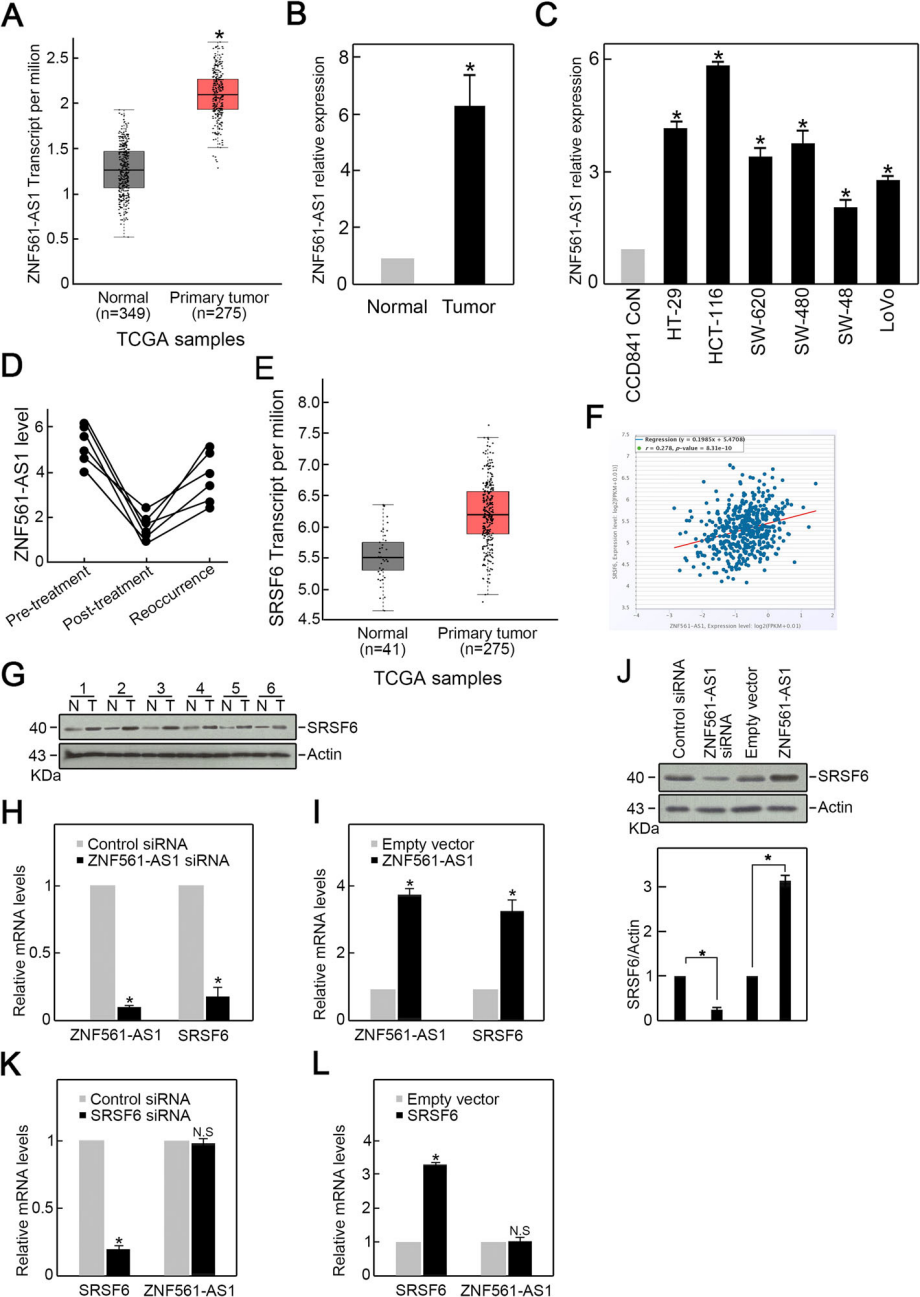
Elevated ZNF561-AS1 positively regulates SRSF6 expression in CRC. **a** ZNF561-AS1 expression in CRC patients. Data from TCGA data resource. *p < 0.05. **b** ZNF561-AS1 expression is elevated in six patient CRC samples. *p < 0.05. **c** ZNF561-AS1 level is elevated in various CRC cell lines relative to CCD841 CoN normal human colonic epithelial cell line. *n* = 3, *p < 0.05. **d** ZNF561-AS1 expression was reduced by chemotherapy but restored in recurrent CRC samples. **e** SRSF6 expression in CRC patients. Data from TCGA data resource. **f** Positive correlation between ZNF561-AS1 and SRSF6 in CRC patients. Data from TCGA data resource. **g** Increased SRSF6 expression was observed in CRC patients tissue samples. **h** ZNF561-AS1 silencing suppressed SRSF6 mRNA levels in HCT-116 cells. *n* = 3, *p < 0.05. **i** ZNF561-AS1 overexpression elevated SRSF6 mRNA levels in HCT-116 cells. *n =* 3, *p < 0.05. **j** ZNF561-AS1 overexpression enhanced SRSF6 proteins level in HCT-116 cells. Actin was employed as loading control. Representative blots are shown (left panel). Ratios of level of SRSF6 vs Actin were computed after densitometric analysis of blot images on Image J (right panel). Data were from 3 independent experiments. **k** SRSF6 knockdown did not alter ZNF561-AS1 expression. *n* = 3, *p < 0.05. **l** SRSF6 overexpression did not alter ZNF561-AS1 expression. *n =* 3, *p < 0.05

To examine the ZNF561-AS1 expression in CRC, analysis of TCGA CRC datasets revealed an elevated ZNF561-AS1 levels in CRC patients (Fig. 1a). Consistently, significantly higher ZNF561-AS1 level was found in our collected CRC patient samples compared to their matched adjacent normal tissue samples (Fig. 1b). Analysis of ZNF561-AS1 level in various CRC cell lines consisting of SW620, HCT-116, HT-29, SW480, SW48 and LoVo cells showed that ZNF561-AS1 expression was increased in all CRC cell lines compared to non-malignant human colon epithelial cell line, CCD841 CoN (Fig. 1c), with HCT-116 expressing the highest ZNF561-AS1 levels. Moreover, we found that compared to CRC samples from untreated patients, samples from patients treated with chemotherapy exhibited lower ZNF561-AS1 levels (Fig. 1d). However, ZNF561-AS1 rose in recurrent CRC samples (Fig. 1d). Together, these data suggest a critical role for ZNF561-AS1 in CRC.

Surprisingly, TCGA CRC dataset analysis found that Serine and arginine-rich splicing factor 6SRSF6 (SRSF6), an oncogenic factor, was also elevated in CRC and showed a positive association with ZNF561-AS1 expression (Fig. 1e-f). Elevated SRSF6 levels were also detected in CRC patients samples (Fig. 1g). To assess the correlation between ZNF561-AS1 and SRSF6 expression in CRC, we used siRNA to silence ZNF561-AS1 or SRSF6 in HCT-116 and then evaluated SRSF6 or ZNF561-AS1 expression. Interestingly, we found that compared to control group, ZNF561-AS1 silencing correlated with reduced SRSF6 mRNA and protein levels (Fig. 1 h, j). While ZNF561-AS1 overexpression correlated with increased SRSF6 mRNA and protein level (Fig. 1 i, j). However, SRSF6 knockdown and overexpression did not affect ZNF561-AS1 expression (Fig. 1 k-l). These data suggested that ZNF561-AS1 was an upstream positive regulator of SRSF6 in CRC cells.

### ZNF561-AS1 sponges miR-26a-5p and miR-128-3p to promote SRSF6 expression

**Fig. 2:**
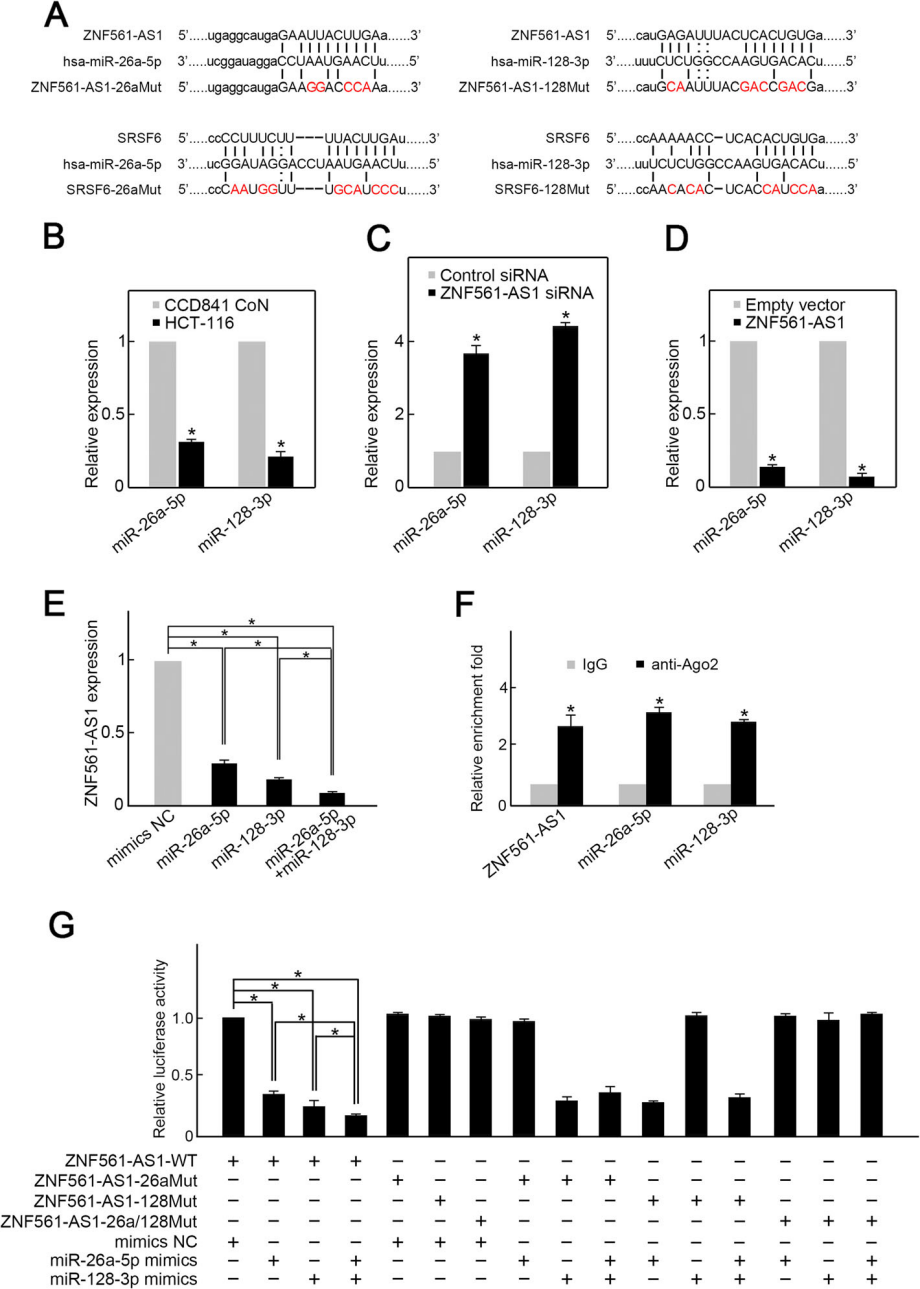
ZNF561-AS1 acts as a ceRNA for miR-26a-5p and miR-128-3p in CRC cells. **a** Schematic of potential miR-26a-5p and miR-128-3p binding sites on ZNF561-AS1. **b** Both miR-26a-5p and miR-128-3p were decreased in HCT-116 cells. *n =* 3, *p < 0.01. **c** ZNF561-AS1 silencing elevated miR-26a-5p and miR-128-3p levels in HCT-116 cells. *n =* 3, *p < 0.05. **d** ZNF561-AS1 overexpression suppressed miR-26a-5p and miR-128-3p levels in HCT-116 cells. *n =* 3, *p < 0.05. **e** Transfection of miR-26a-5p or/and miR-128-3p mimics suppressed ZNF561-AS1 expression. *n =* 3, *p < 0.05. **f** Relative enrichments of ZNF561-AS1, miR-26a-5p, as well as miR-128-3p in RISC was examined by RIP assay using anti-Ago2 antibody. *n* = 3, *p < 0.01. **g** Luciferase reporter assay. HCT-116 cells were transfected as indicated. Relative luciferase activity was assessed 48 h post transfection. *n =* 3, *p < 0.01

LncRNAs modulate gene transcription by functioning as miRNA sponges [18]. Using Starbase and Targetscan bioinformatics analysis, we surprisingly found that ZNF561-AS1 and SRSF6 bear potential binding sites for miR-26a-5p, as well as miR-128-3p (Fig. 2a). Thus, we hypothesize that ZNF561-AS1 might enhance SRSF6 expression by acting as a sponge of miR-26a-5p and miR-128-3p. To test this possibility, we first examined miR-26a-5p and miR-128-3p expression in HCT-116 cells and demonstrated that the levels of miR-128-3p and miR-26a-5p were remarkably reduced in HCT-116 cells in comparison of CDD841 CoN cells (Fig. 2b). Moreover, ZNF561-AS1 silencing upregulated levels of miR-26a-5p and miR-128-3p, while its overexpression reduced levels of miR-26a-5p and miR-128-3p (Fig. 2 c-d). In contrast, miR-26a-5p mimics and/or miR-128-3p mimics suppressed ZNF561-AS1 expression (Fig. 2e). These observations indicating a reciprocal regulation between ZNF561-AS1 and miR-26a-5p/miR-128-3p. Next, we used RIP analysis to determine whether ZNF561-AS1 and miR-26a-5p/miR-128-3p occupy the same RISC complex. To this end, HCT-116 lysates were immunoprecipitated using anti-Ago2 antibody and enrichment of ZNF561-AS1, miR-26a-5p, and miR-128-3p in immunoprecipitates were assessed by RT-qPCR. We found that the levels of ZNF561-AS1, miR-26a-5p, and miR-128-3p were all remarkably higher in Ago2 pellets relative to IgG pellets (Fig. 2f). Next, luciferase reporter assays to determine if ZNF561-AS1 was a direct target of miR-26a-5p and miR-128-3p demonstrated that miR-26a-5p or/and miR-128-3p mimics remarkably reduced ZNF561-AS1-WT luciferase activity but not that of single or double binding site mutants (Fig. 2g). Altogether, these data suggest that ZNF561-AS1 is a ceRNA for miR-26a-5p and miR-128-3p.

**Fig. 3:**
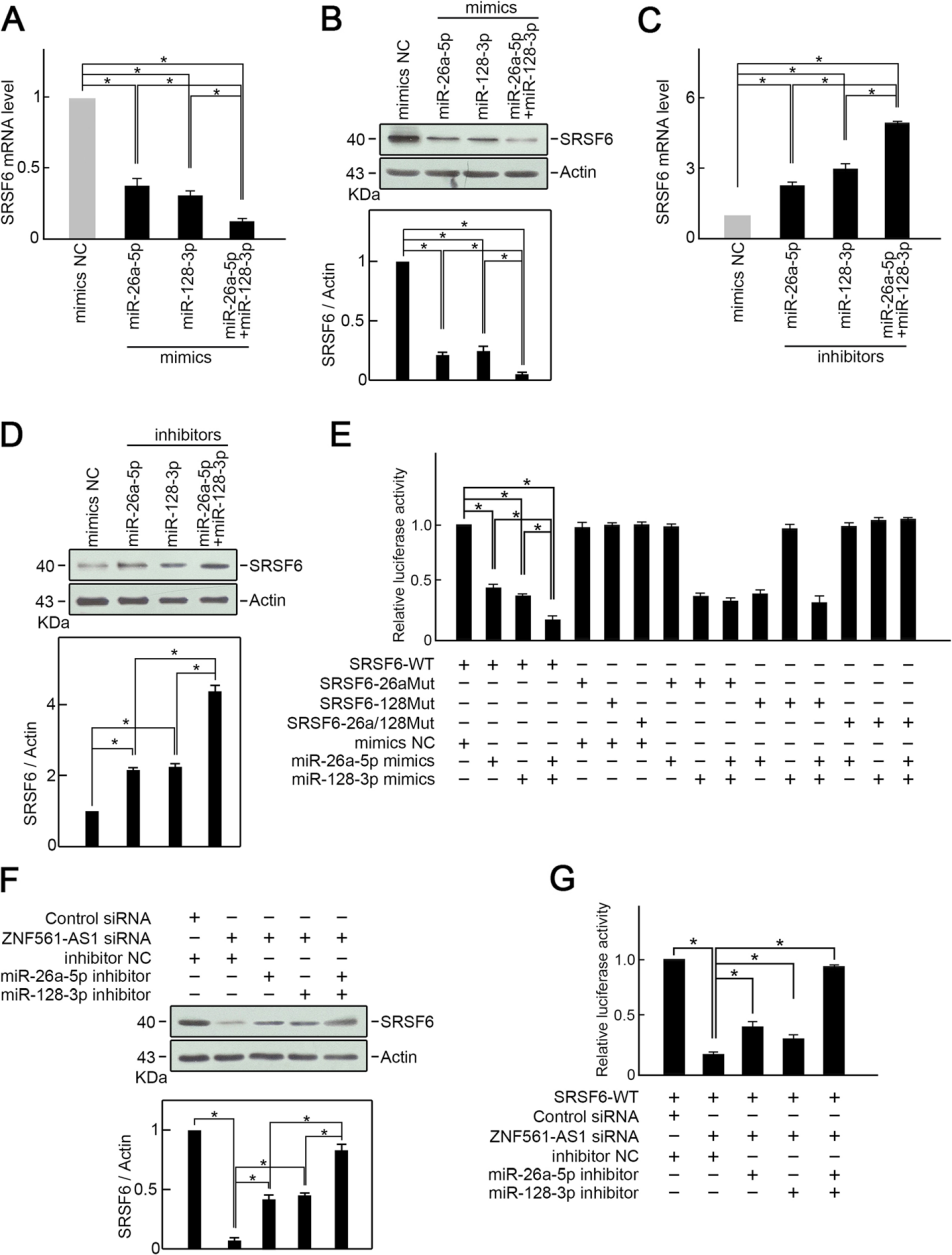
ZNF561-AS1 promotes SRSF6 expression by sponging miR-26a-5p and miR-128-3p. **a** Transfection of miR-26a-5p or/and miR-128-3p mimics suppressed SRSF6 mRNA levels. *n* = 3, *p < 0.01. **b** Transfection of miR-26a-5p or/and miR-128-3p mimics suppressed SRSF6 protein level. Actin was employed as loading control. Representative blots are shown (upper panel). Ratios of SRSF6 level vs Actin were determined after densitometric analysis of blot images on Image J (lower panel). Data were from 3 independent experiments. *p < 0.01. **c** Knockdown of miR-26a-5p or/and miR-128-3p increased SRSF6 mRNA level. *n =* 3, *p < 0.01. **d** Knockdown of miR-26a-5p or/and miR-128-3p increased SRSF6 protein level. Actin was served as loading control. Representative blots were shown (upper penal). Ratios of level of SRSF6 vs Actin were calculated after densitometric analysis of blot images using NIH Image J 1.61 (lower panel). Data were from three individual experiments. *p < 0.05. **e** Luciferase reporter assay. HCT-116 cells were transfected as indicated. Relative luciferase activity was explored 48 h post-transfection. *n* = 3, *p < 0.05. **f** MiR-26a-5p or/and miR-128-3p silencing restored SRSF6 expression in ZNF561-AS1 depleted HCT-116 cells. Actin served as loading control. Representative blots are shown (upper panel). Ratios of SRSF6 vs Actin levels were computed after densitometric analysis of blot images on Image J (lower panel). Data were from 3 independent experiments. *p < 0.01. **g** Luciferase reporter assay. MiR-26a-5p or/and miR-128-3p silencing restored SRSF-WT luciferase activity in ZNF561-AS1-depleted HCT-116 cells. *n* = 3, *p < 0.01

Next, we examined whether miR-26a-5p and miR-128-3p suppress SRSF6 expression through potential binding sites. Overexpression of miR-26a-5p or miR-128-3p suppressed SRSF6 expression (Fig. 3 a-b). Moreover, simultaneous overexpression of miR-26a-5p and miR-128-3p suppressed SRSF6 expression even more strongly than miR-26a-5p or miR-128-3p alone (Fig. 3 a-b). MiR-26a-5p and/or miR-128-3p silencing enhanced SRSF6 expression (Fig. 3 c-d). Luciferase reporter assay illustrated that miR-26a-5p and/or miR-128-3p mimics remarkably suppressed SRSF6-WT luciferase activity but not that of single or double binding site mutants (Fig. 3e).

Since we had already demonstrated that ZNF561-AS1 is an upstream SRSF6 regulator, we then assessed if ZNF561-AS1 regulates SRSF6 expression via miR-26a-5p and/or miR-128-3p. Evaluation of the expression of SRSF6 in HCT-116 cells co-transfected with ZNF561-AS1 siRNA and miR-26a-5p and/or miR-128-3p inhibitors found that in ZNF561-AS1 depleted cells, single miR-26a-5p inhibitor or miR-128-3p inhibitor partially reversed SRSF6 expression (Fig. 3f), whereas co-transfection with miR-26a-5p and miR-128-3p inhibitors almost fully rescued SRSF6 expression (Fig. 3f). Similar results were obtained from luciferase reporter assays. ZNF561-AS1 silencing markedly suppressed SRSF6-WT luciferase activity (Fig. 3g). However, miR-26a-5p and/or miR-128-3p inhibitors could partially or fully rescue luciferase activity in ZNF561-AS1 depleted cells (Fig. 3g). Taken together, these data show that ZNF561-AS1 promotes SRSF6 expression in CRC cells by sponging miR-26a-5p and miR-128-3p.

### ZNF561-AS1 is critical for CRC cells proliferation and survival

**Fig. 4:**
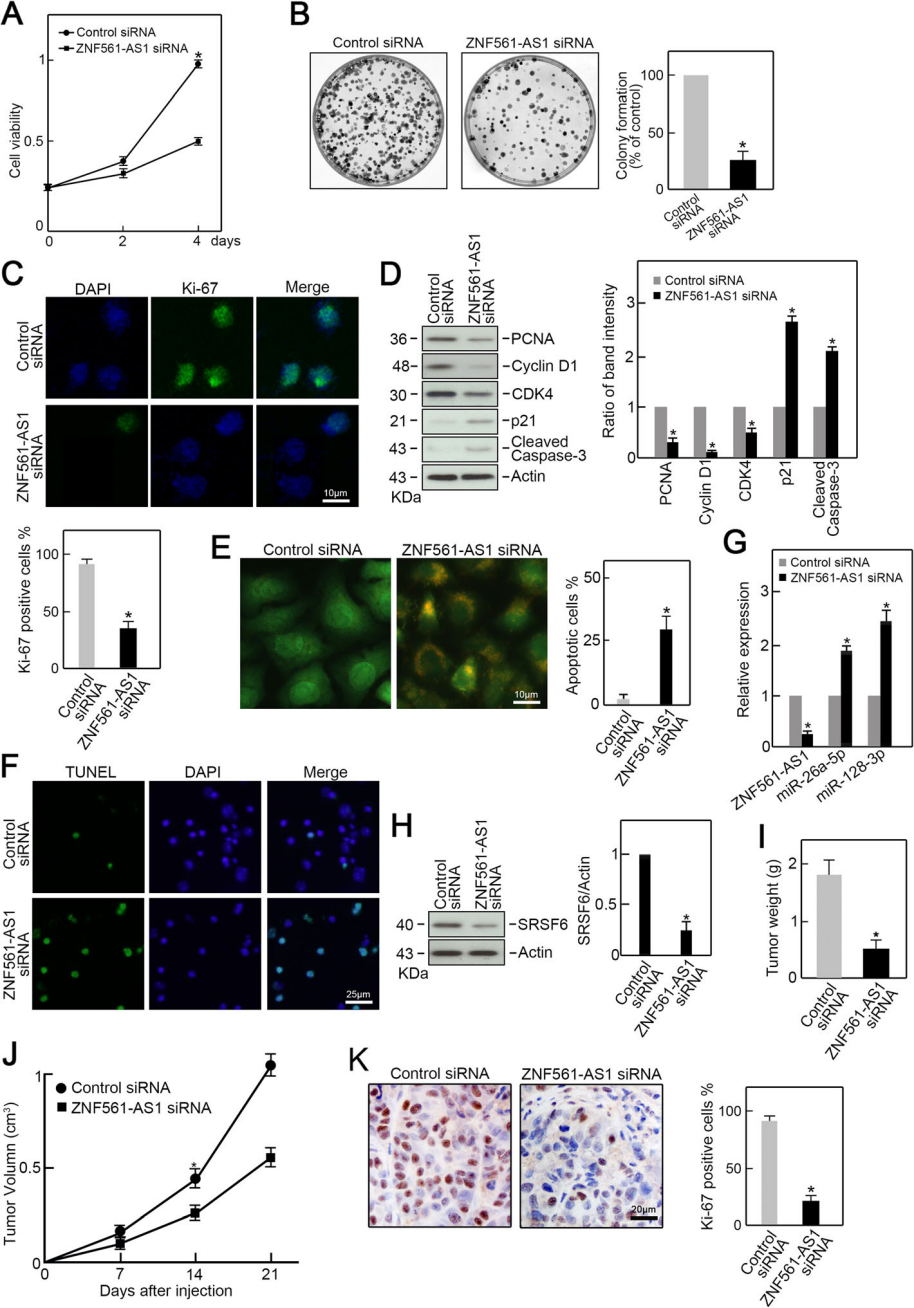
ZNF561-AS1 silencing suppresses CRC progression in vitro and in vivo. **a** ZNF561-AS1 silencing inhibited HCT-116 growth. *n =* 3, *p < 0.01. **b** Colony formation assay. ZNF561-AS1- or mock-silenced HCT-116 cells were planted in 6 cm dishes and colonies counted after 12 days. Formed colonies were stained with crystal violet and counted. *n =* 3, *p < 0.05. **c** Ki-67 level was significantly reduced in ZNF561-AS1 depleted cells compared to controls assessed by immunofluorescence staining. *n =* 3, *p < 0.01. **d** PCNA, Cyclin D1, CDK4, p21, and cleaved caspase-3 levels in ZNF561-AS1- and mock-silenced HCT-116 cells. Actin served as loading control. Representative blots are shown (left panel). Ratios of PCNA, Cyclin D1, CDK4, p21, and cleaved caspase-3 vs Actin levels were computed after densitometric analysis of blot images on Image J (right panel). Data are 3 independent experiments. *p < 0.05. **e** Knockdown of ZNF561-AS1 induced cell apoptosis assessed by AO/EB staining. *n* = 3, *p < 0.01. **f** Knockdown of ZNF561-AS1 induced cell apoptosis assessed by TUNLE staining. *n* = 3. **g** The expression of ZNF561-AS1, miR-26a-5p and miR-128-3p in xenograft tumors. *n =* 3, *p < 0.05. **h** Levels of SRSF6 in xenograft tumors. Actin was served as loading control. Representative blots were shown (left penal). Ratios of level of SRSF6 vs Actin were calculated after densitometric analysis of blot images using NIH Image J 1.61 (right panel). Data were from three individual experiments. *p < 0.01. **i** Knockdown of ZNF561-AS1 reduced xenograft tumors weight. *n =* 3, *p < 0.05. **j** Knockdown of ZNF561-AS1 reduced xenograft tumors volume. *n =* 3, *p < 0.05. **k** Ki-67 immunohistochemical staining in xenograft tumor specimens. *n =* 3, *p < 0.05

Since we found that ZNF561-AS1 is dramatically increased in CRC and it positively regulates SRSF6 expression, we next examined its biological effects on CRC cells. As expected, we found ZNF561-AS1 silencing significantly suppressed HCT-116 growth and colony formation compared to controls (Fig. 4 a-b). Ki-67 Immunofluorescence staining exhibited significantly lower Ki-67 signal in ZNF561-AS1 depleted HCT-116 cells compared to controls, indicating that ZNF561-AS1 silencing suppressed HCT-116 cells proliferation (Fig. 4c). Additionally, we found that expression of the cell cycle promoting factors, PCNA, CDK4, and Cyclin D1 was reduced markedly in ZNF561-AS1 deficient HCT-116 cells (Fig. 4d). However, p21, a strong cell cycle inhibitor, was elevated (Fig. 4d). Interestingly, the level of cleaved caspase-3, an apoptotic factor, was increased upon ZNF561-AS1 knockdown (Fig. 4d). This observation led us to examine whether ZNF561-AS1 silencing would trigger cell apoptosis in HCT-116 cells. AO/EB staining revealed that knockdown of ZNF561-AS1 increased the proportion of apoptotic cells in ZNF561-AS1 depleted HCT-116 cells compared to control cells (Fig. 4e). This phenotype was further confirmed by TUNEL staining (Fig. 4f).

To study the role of ZNF561-AS1 in vivo, ZNF561-AS1 siRNA or control siRNA transfected HCT-116 cells were subcutaneously injected into the flanks of nude mice. After 3 weeks, the mice were sacrificed, and xenograft tumors were isolated. RT-qPCR analysis confirmed ZNF561-AS1 depletion in tumors from ZNF561-AS1 depleted cells (Fig. 4g), which also exhibited increased miR-26a-5p and miR-128-3p levels and reduced SRSF6 levels relative to controls (Fig. 4 g-h). Tumors from ZNF561-AS1 depleted cells were smaller in size and lower in weight compared to control tumors. (Fig. 4 i-j). Immunohistochemical staining revealed lower Ki-67 levels in ZNF561-AS1 depleted tumors (Fig. 4k). Together, these data indicate that ZNF561-AS1 is crucial for CRC cell proliferation, as well as survival both in vitro and in vivo.

### ZNF561-AS1 promotes CRC cells proliferation via SRSF6

**Fig. 5:**
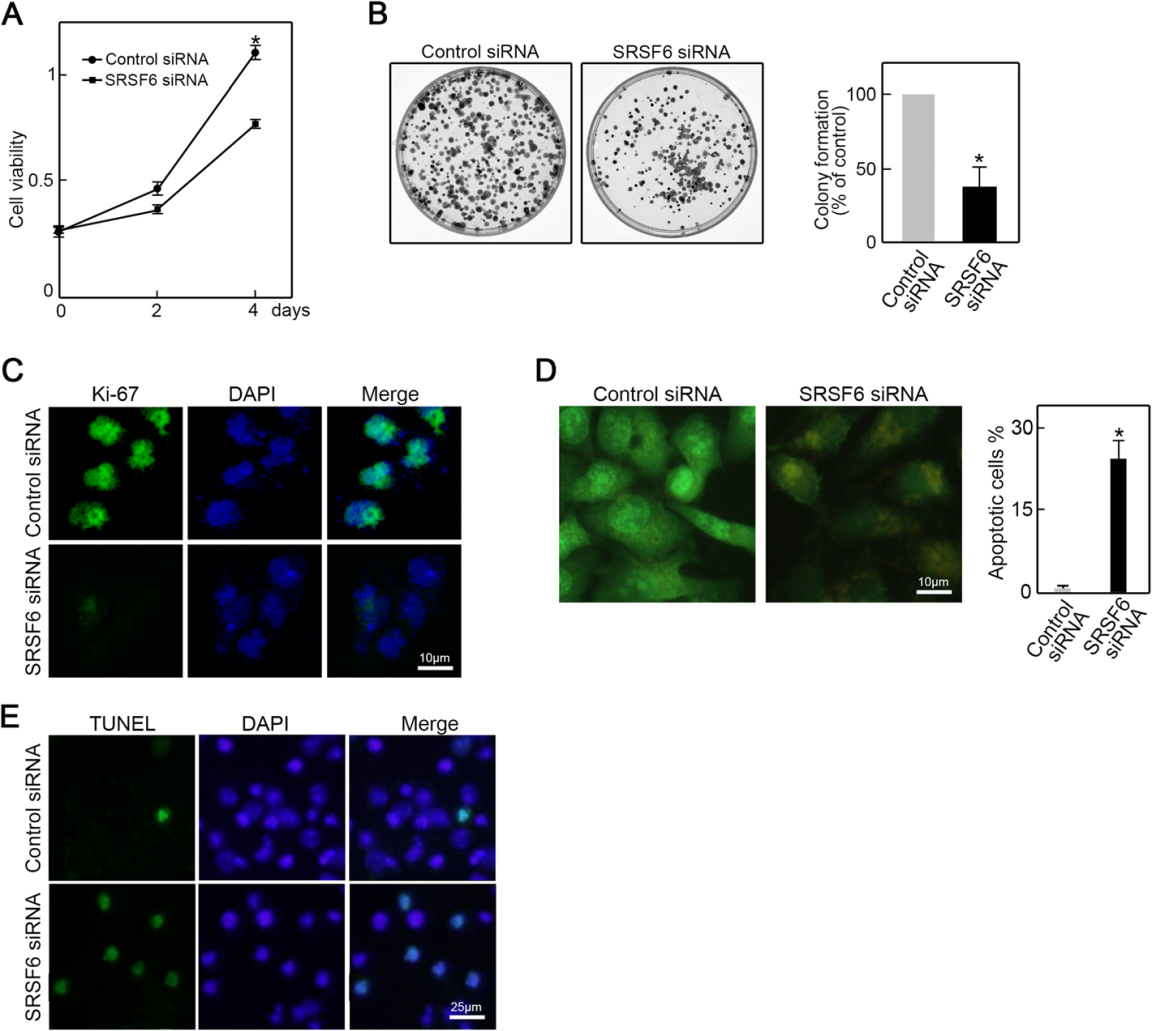
RSF6 silencing repressed cell proliferation and triggered apoptosis in CRC cells. **a** Knockdown of SRSF6 inhibited HCT-116 cells growth. *n =* 3, *p < 0.01. **b** Colony formation assay. HCT-116 cells transfected with SRSF6 siRNA or control siRNA were seeded in 6 cm dishes for 12 days culture. Formed colonies were stained with crystal violet and counted. *n =* 3, *p < 0.05. **c** Proliferation capabilities of ZNF561-AS1 siRNA or control siRNA transfected HCT-116 cells was examined by Ki-67 immunofluorescence staining. *n =* 3. **d** Knockdown of SRSF6 induced cell apoptosis assessed by AO/EB staining. *n =* 3, *p < 0.01. **e** Knockdown of SRSF6 induced cell apoptosis assessed by TUNLE staining. *n =* 3

SRSF6 is associated with the progression of various cancers. Here, we find that SRSF6 is essential for CRC cell proliferation and survival as its depletion phenocopies ZNF561-AS1 knockdown as revealed by CCK-8 analysis, colony formation, and Ki-67 immunofluorescence staining (Fig. 5 a-c). Moreover, AO/EB and TUNEL assay demonstrated that SRSF6 depletion triggered apoptosis in HCT-116 cells (Fig. 5 d-e). These data show that SRSF6 is crucial for CRC proliferation and survival. Depletion of SRSF6 induced similar phenotypes as ZNF561-AS1 knockdown.

**Fig. 6:**
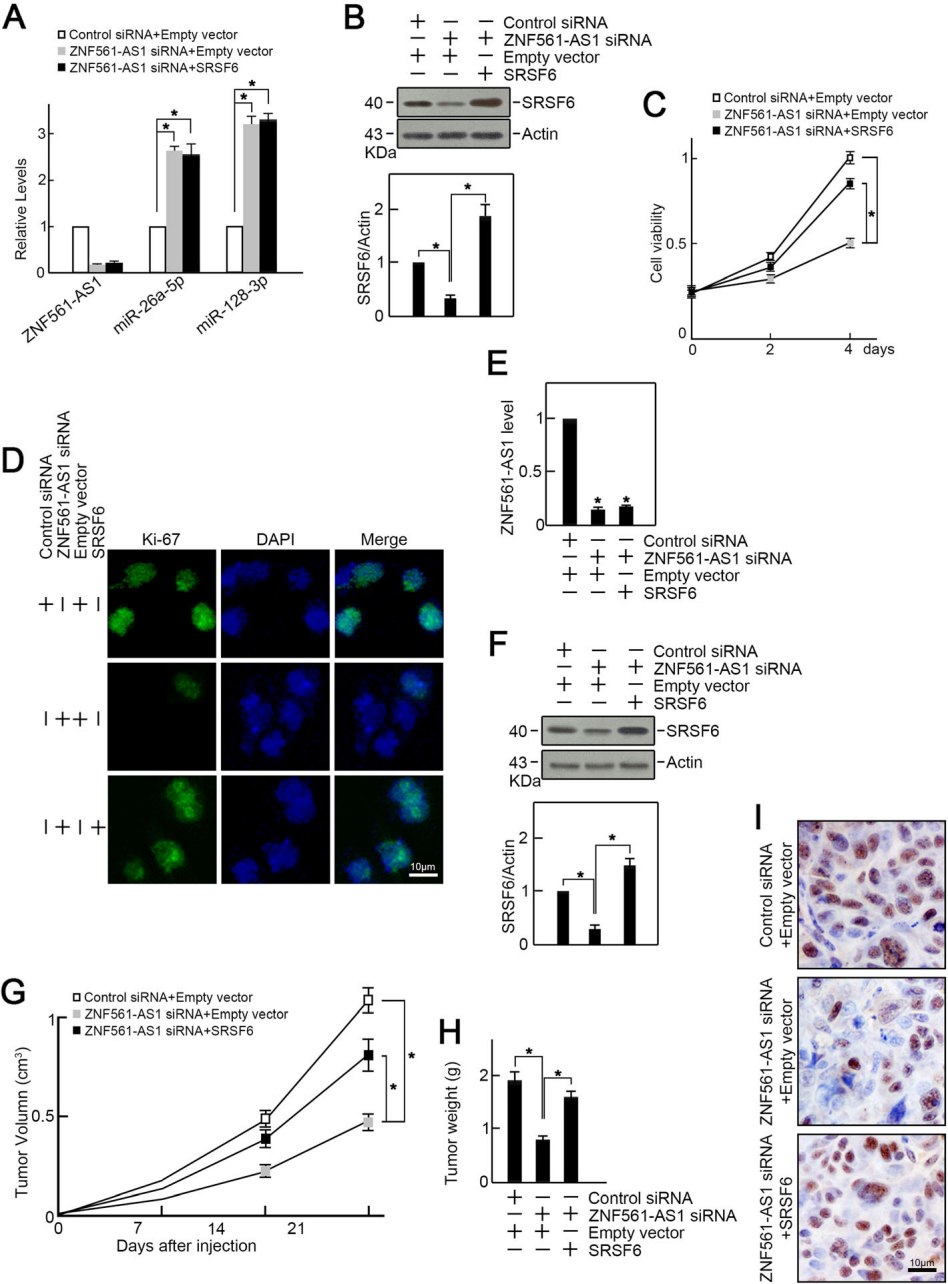
Exogenous SRSF6 expression rescued proliferation of ZNF561-AS1-depleted CRC cells in vitro and in vivo. HCT-116 cells were co-transfected as indicated. **a** RT-qPCR analysis of ZNF561-AS1, miR-26a-5p, and miR-128-3p levels. *n =* 3, *p < 0.05. **b** SRSF6 expression was examined by immunoblotting. Actin served as loading control. Representative blots were shown (upper penal). Ratios of level of SRSF6 vs Actin were calculated after densitometric analysis of blot images using NIH Image J 1.61 (lower panel). Data were from three individual experiments. *p < 0.01. **c** Enforced expression of SRSF6 restored cell growth capabilities in HCT-116 cells depleted of ZNF561-AS1. *n =* 3, *p < 0.05. **d** Enforced expression of SRSF6 restored Ki-67 expression in HCT-116 cells depleted of ZNF561-AS1. *n =* 3. **e** ZNF561-AS1 expression in xenograft tumors. *n =* 3, *p < 0.01. **f** Co-transfection of SRSF6 expression vector restored SRSF6 level in xenograft tumors. Actin served as loading control. *n =* 3, *p < 0.01. **g** Exogenous expression of SRSF6 rescued tumor volume. *n =* 3, *p < 0.05. **h** Exogenous expression of SRSF6 rescued tumor weight. *n =* 3, *p < 0.05. **i** SRSF6 overexpression restored Ki-67 levels in xenograft tumors. *n =* 3

To further test whether ZNF561-AS1 promoted CRC cell proliferation via SRSF6, we overexpressed SRSF6 in ZNF561-AS1 depleted HCT-116 cells. We found that ZNF561-AS1 expression was suppressed by ZNF561-AS1 siRNA transfection (Fig. 6a) and the levels of miR-26a-5p and miR-128-3p were increased (Fig. 6a) while the level of SRSF6 was decreased (Fig. 6b). Co-transfection of SRSF6 expression vector restored SRSF6 expression in ZNF561-AS1 depleted HCT-116 cells and as expected, rescued proliferation in ZNF561-AS1-depleted cells as revealed by CCK-8 and Ki-67 assays (Fig. 6 c-d).

Furthermore, HCT-116 cells depleted of ZNF561-AS1 were co-transfected with SRSF6 and subcutaneously injected into the flanks of nude mice. After 3 weeks, all mice were sacrificed, and xenograft tumors were isolated. qRT-PCR demonstrated that ZNF561-AS1 siRNA suppressed the level of ZNF561-AS1 in xenograft tumors (Fig. 6e). SRSF6 overexpression restored SRSF6 expression in ZNF561-AS1 depleted tumors (Fig. 6f). ZNF561-AS1 depleted tumors were smaller in size and lower in weight, compared to controls (Fig. 6 g-h). But ectopic SRSF6 expression rescued this phenotype (Fig. 6 g-h). Consistently, immunohistochemical staining analysis revealed reduced Ki-67 levels in ZNF561-AS1 depleted tumors compared to controls, which was restored by SRSF6 overexpression (Fig. 6 i). Collectively, these data indicate that ZNF561-AS1 promotes CRC cells proliferation by modulating SRSF6 expression.

## Discussion

Herein, we investigated the role of ZNF561-AS1 in CRC and the reasonable mechanism. ZNF561-AS1 was upregulated in CRC compared to matched adjacent normal tissues and ZNF561-AS1 silencing suppressed CRC cells proliferation and survival, suggesting an oncogenic role in CRC. In contrast with our findings, another study found that ZNF561-AS1 downregulation promoted migration and invasion of laryngeal cancer cells (LSCC), indicating tumor suppressor function [19]. This difference was mostly considered from the different histological types [20]. The disparity in ZNF561-AS1 function in LSCC vs CRC suggests tissue-specific or cancer-specific effects.

Increasing evidence shows that numerous lncRNAs are dysfunctional in CRC, acting as oncogenes or tumor suppressors [21, 22]. LncRNA CCAL regulates CRC progression by targeting the activator protein 2α (AP-2α), which activates Wnt/β-catenin signaling [23]. LncRNA DLEU1 contributes to CRC progression by activating KPNA3 via SMARCA1 recruitment, which is an essential subunit of the NURF chromatin remodeling complex [24]. LncRNA SNHG5 enhances CRC cell survival via counteracting STAU1-induced mRNA destabilization [25]. Our data indicate that ZNF561-AS1 modulates CRC cell proliferation along with survival. In vitro and in vivo studies indicate that ZNF561-AS1 sequesters miR-26a-5p and miR-128-3p, thereby enhancing SRSF6 expression and promoting CRC cell proliferation and survival.

SRSF6 is a member of the serine-arginine-rich splicing factor family. These proteins are essential for pre-mRNA splicing and mRNA stability, export, and translation [26]. SRSF6 is a proto-oncogene often overexpressed in human skin cancer [27]. By controlling exon skipping, SRSF6 is critical for leukemia cells survival [28]. SRSF6 is also overexpressed in CRC [29]. Evidence show that SRSF6-regulated alternative splicing of ZO-1 promotes CRC progression by directly binding its motif to ZO-1 exon23 [30]. This alternative splicing effect relies on SRSF6’s RRM2 domain. Here, we find that ZNF561-AS1 promotes SRSF6 expression by acting as sponge of miR-26a-5p and miR-128-3p. SRSF6 overexpression rescued proliferation and survival in ZNF561-AS1 depleted cells. Indicating that ZNF561-AS1 regulates CRC cells proliferation and survival by modulating SRSF6 expression. Because SRSF6 knockdown did not alter ZNF561-AS1 expression, while ZNF561-AS1 knockdown suppressed SRSF6 expression, we speculate that ZNF561-AS1 is an upstream post-transcriptional regulator of SRSF6.

## Conclusion

In summary, our study revealed an essential role of over-expression of ZNF561-AS1 for CRC cells proliferation and survival. ZNF561-AS1 servers as a ceRNA, competitively binding to miR-26a-5p and miR-128-3p to enhance the expression of SRSF6, an alternative splicing factor. These findings provide novel insight into our understanding of CRC progression and highlight ZNF561-AS1 as a potential therapeutic target against CRC.

## Supplementary Information

**Additional file 1: Table S1.** CRC patient information.

## Data Availability

The data used and analyzed during the current study are available from the corresponding author on reasonable request.

## Abbreviations

CRC: Colorectal cancer
ZNF561-AS1: ZNF561 antisense RNA 1
RIP: RNA binding protein immunoprecipitation
TCGA: The Cancer Genome Atlas
LncRNA: Long non-coding RNA
TUNEL: Terminal deoxynucleotidyl transferase dUTP nick end labeling
SRSF6: Serine and arginine-rich splicing factor 6
ANOVA: Analysis of variance
