# Supplementary figures and images for: Oncogenic lncRNA ZNF561-AS1 is essential for colorectal cancer proliferation and survival through regulation of miR-26a-3p/miR-128-5p-SRSF6 axis
Source: J Exp Clin Cancer Res. 2021 Feb 23;40:78. doi: 10.1186/s13046-021-01882-1 (PMC7903733; doi:10.1186/s13046-021-01882-1)

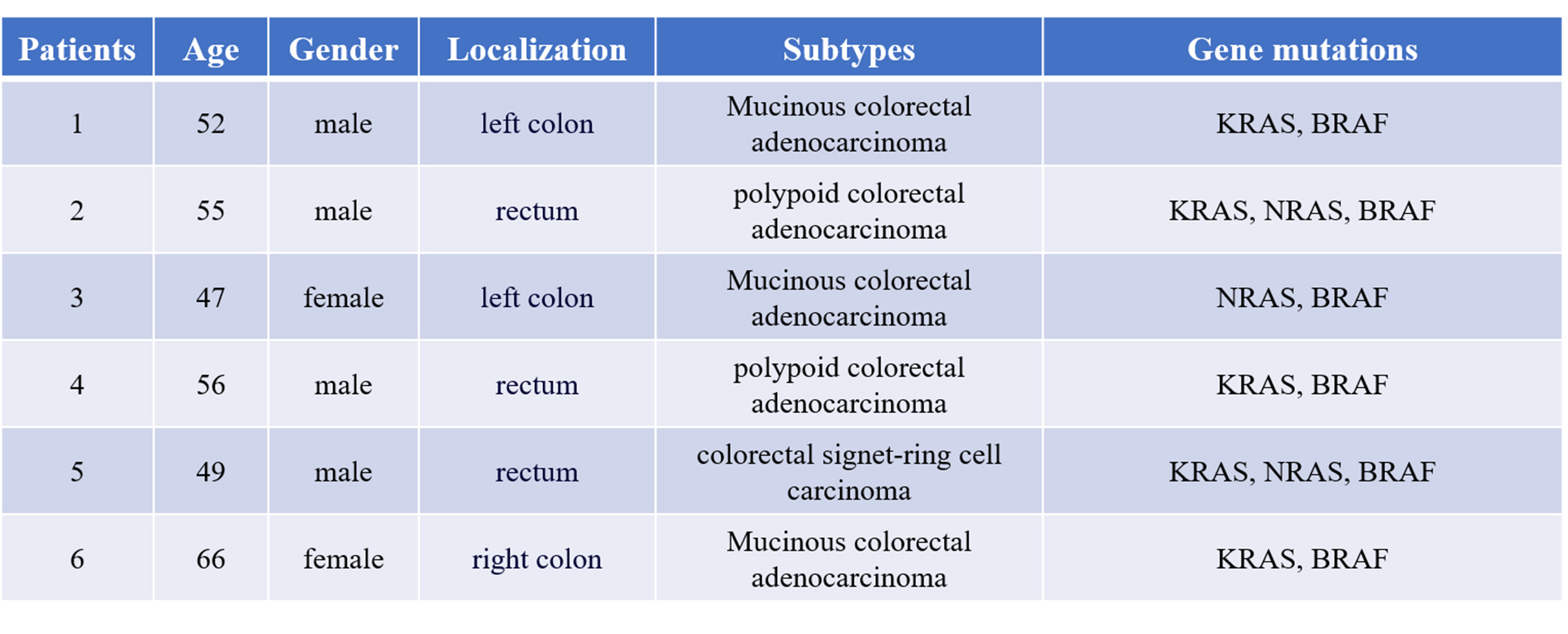

Supplement: Supplementary file 1 — Additional file 1: Table S1. CRC patient information. [file 13046_2021_1882_MOESM1_ESM.jpg]
